# Hot tub lung machine

**DOI:** 10.1002/rcr2.682

**Published:** 2020-11-11

**Authors:** Matthew Corbitt, David Deller

**Affiliations:** ^1^ Department of Medicine Cairns and Hinterland Hospital and Health Service Cairns QLD Australia; ^2^ School of Medicine Griffith University Gold Coast QLD Australia; ^3^ Gold Coast Respiratory & Sleep Clinic Pindara Private Hospital Gold Coast QLD Australia

**Keywords:** Hot tub lung, hypersensitivity pneumonitis, non‐tuberculous mycobacteria

## Abstract

Hot tub lung (HTL) is a pulmonary disease where a hypersensitivity pneumonitis is caused by exposure and inhalation of aerosolized non‐tuberculous mycobacteria (NTM) traditionally from hot water steam. HTL can affect both immunocompromised and healthy individuals, and diagnosis is dependent on high clinical suspicion in conjunction with high‐resolution computed tomography (HRCT) and microbiological evidence. We present, to our knowledge, the only case of HTL occurring from an indoor swimming pool affecting five members of a family, in which one member was not a resident of the household.

## Graphical abstract

Hot tub lung (HTL) is a pulmonary disease where a hypersensitivity pneumonitis is caused by exposure and inhalation of aerosolized non‐tuberculous mycobacteria (NTM) traditionally from hot water steam. We present, to our knowledge, the only case of HTL occurring from an indoor swimming pool affecting five members of a family, in which one member was not a resident of the household.

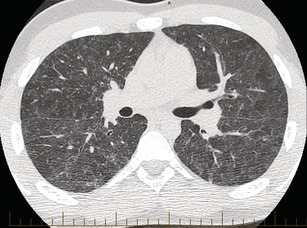

## Introduction

This case demonstrates hot tub lung (HTL) secondary to aerosolized non‐tuberculous mycobacteria (NTM) from an indoor swimming pool in five members of a family.

## Case Report

A 17‐year‐old man was admitted after presenting to the emergency department 10 days post ankle reconstruction, less than seven days since discharge, with cough, low‐grade fevers, and progressively worsening dyspnoea. On presentation, there was tachypnoea with exercise‐induced hypoxaemia (peripheral capillary oxygen saturation (SpO_2_): 82%) on mobilizing short distances. No viral symptoms or other systemic symptoms including recent rashes, joint pain, or haematuria were reported. There was no prior medical or respiratory history. There was also no recent travel history or sick contacts; however, his younger brother (aged 14) had recent cough associated with dyspnoea and his mother reported having a persistent cough over the preceding three months.

On examination, there were extensive crackles bilaterally on chest auscultation without additional signs of heart failure. Supplemental oxygen was required via nasal cannula at 3 L/min to maintain oxygen saturations above 90%.

Additional investigations showed mild neutrophilia (8.6 × 10^9^/L), eosinophilia (1.17 × 10^9^/L), and a mildly elevated C‐reactive protein (10 mg/L). An extensive search for infectious causes was undertaken, including blood cultures, respiratory virus polymerase chain reaction (PCR), urinary *Legionella* and *Streptococcus pneumoniae* antigens, *Mycoplasma* IgM, *Chlamydia psittaci* and *Chlamydia pneumoniae* IgG, and *Aspergillus* and avian precipitin studies, which were all negative. An autoimmune screen was also negative.

Pulmonary function tests demonstrated a restrictive pattern of impairment with significant reductions in forced expiratory volume in 1 sec (FEV_1_) of 2.52 L (64% predicted), forced vital capacity (FVC) of 3.42 L (72% predicted) without bronchodilator reversibility, and diffusing capacity of carbon monoxide (DLCO) of 19.54 mL/min/mmHg (67% predicted).

**Figure 1:**
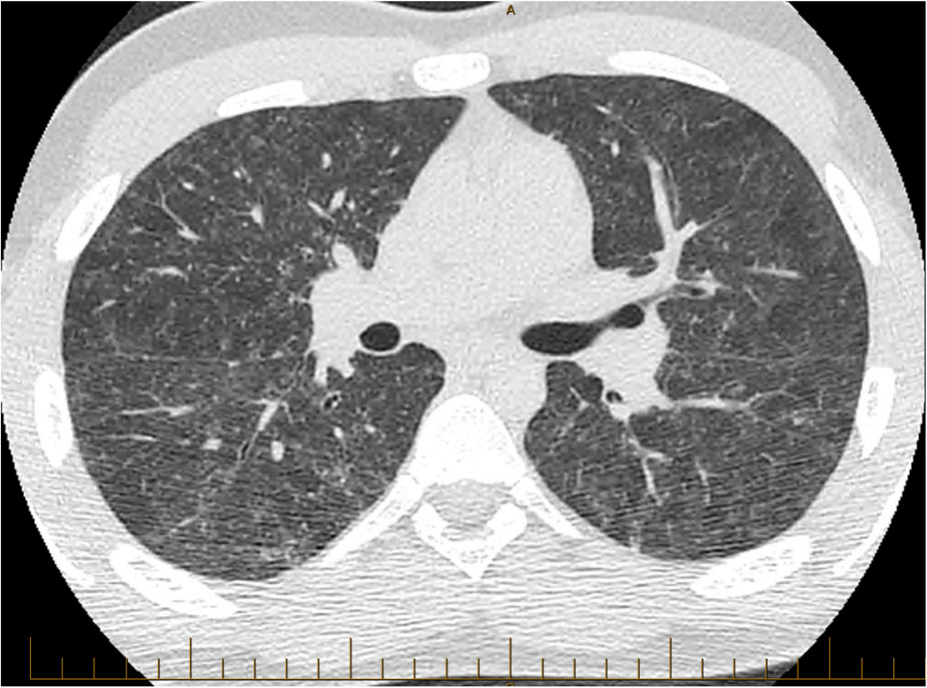
Computed tomography (CT) scan of the patient at diagnosis demonstrating bilateral, symmetrical, diffuse patchy ground‐glass opacities with associated small volume adenopathy, consistent with hypersensitivity pneumonitis (HP)‐associated non‐tuberculous mycobacteria (NTM) infection.

A computed tomography (CT) scan of the chest demonstrated bilateral, symmetrical, diffuse patchy ground‐glass opacities with associated small volume adenopathy; however, no micronodularity was seen (Fig. 1).

Further questioning identified that the family's indoor swimming pool had recently transitioned from chlorine‐based sanitation to a non‐chlorine based (Perox and hybrid ozone) infiltration system. An occupational hygienist attended the family home and identified that mould (*Scedosporium* spp.) was present in the house due to excess humidity, and that the family had a pet cockatiel which resided in their kitchen area. Additionally, microscopic analysis further identified another mould (*Cladosporium* spp.). Considering the environmental antigens present in the family home in conjunction with the patient's clinical course, a preliminary diagnosis of hypersensitivity pneumonitis (HP) was made. The family reported no other potential exposures to other water sources that could have precipitated this diagnosis.

Further testing for HP (*Micropolyspora faeni*, terrestrial actinomycetes, and pigeon serum IgG; *Penicillium*; *Cladosporium*; *Candida albicans*; *Alternaria*; and *Setomelanomma*) was negative. Elevation in both specific IgE and IgG *Aspergillus* antibodies was not seen.

A bronchoalveolar lavage (BAL) for cell count, differential, standard, and acid‐fast bacilli culture demonstrated lymphocytosis (36%) without eosinophilia (0%). Standard culture did not grow pathogens.

Corticosteroids were withheld after daily improvement with supportive care allowing discharge to a temporary residence. Environmental testing of the pool water was undertaken via microscopy and nucleic acid amplification testing (NAAT) of serial water samples with the assistance of the Mycobacterial Reference Laboratory at the Royal Brisbane and Women's Hospital, as per government protocols. Other main water sources in the house (kitchen, bathrooms, and showers, including faucets) were also tested.

*Mycobacterium intracellulare* was subsequently cultured from multiple washings of the patient's BAL sample, as well as the indoor pool sample, thus supporting the diagnosis of HTL. *Mycobacterium avium* and *Mycobacterium marinum* were also isolated from the pool water, but not the house water. The remaining water sources all returned negative results, except for the ensuite shower, which grew *Mycobacterium lentiflavum*. No other analysis (e.g. restriction fragment length polymorphism fingerprinting or other) was undertaken.

Commercial cleaning of the property and disinfection of the pool was undertaken as per the local health authority's “Responding to Unsatisfactory Water Sample Results Procedure,” which included: pasteurization, chemical cleaning, superchlorination, and residual chlorination.

**Figure 2:**
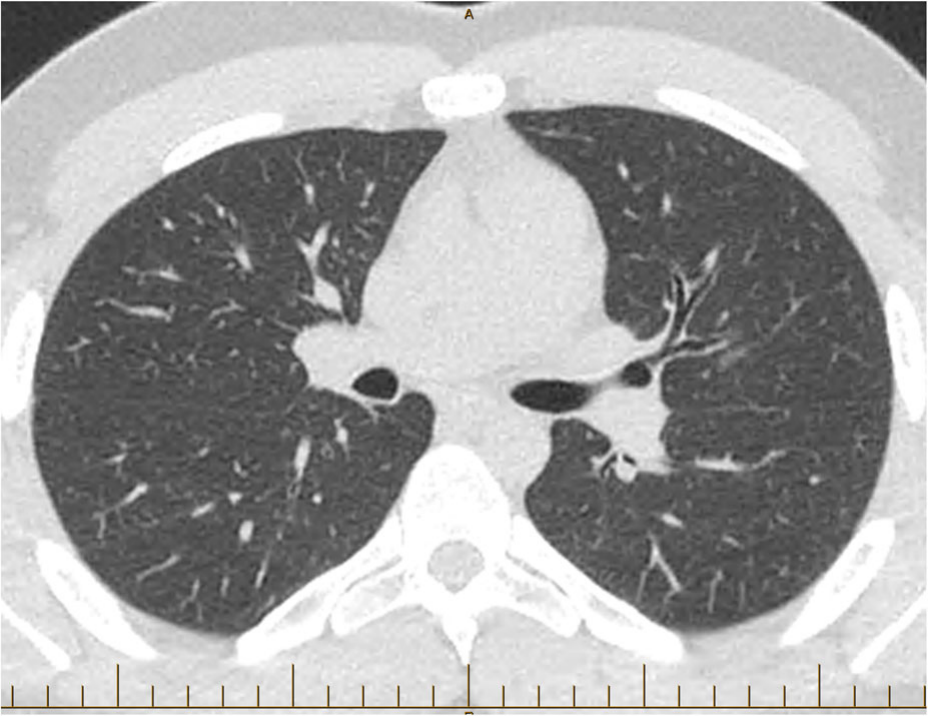
High‐resolution computed tomography (HRCT) scan of the patient at two years follow‐up demonstrating resolution of the prior changes.

Antimicrobial therapy was not required and close follow‐up in conjunction with the removal of the environmental antigens was the mainstay of treatment. The patient's lung function tests improved and are now stable at low normal values (FEV_1_: 3.98 L, 88% predicted; FVC: 4.96 L, 93% predicted; DLCO: 27.16, 74% predicted). Additionally, a high‐resolution CT (HRCT) scan performed at two years was within normal limits (Fig. 2).

Interestingly, both of the patient's parents, younger brother, and grandmother all developed a persistent cough with varying degrees of dyspnoea and had gas transfer values of 50–60% predicted with radiological changes consistent with NTM disease, including some subpleural nodular changes in the patient's younger brother and grandmother's CT. His mother also developed sweats in addition to class I dyspnoea. Furthermore, she had BAL and biopsies performed, which did not isolate any *Mycobacterium*; however, well‐defined interstitial granulomatous inflammation was identified with areas of punctate necrosis, a pattern also seen in mycobacterial infection.

All other investigations for the family were unremarkable and they were treated conservatively in light of their normal immune function. Intriguingly, the grandmother did not live at the family's residence but visited frequently. At two‐year follow‐up, none of the family members were experiencing any residual respiratory symptoms, although pulmonary function tests demonstrated persistent mild reduction in DLCO.

It is believed that the family's symptoms were present for months, however, the patient's symptoms deteriorated acutely following post‐operative home convalescence when he spent substantial time in a media room directly adjacent to the pool.

## Discussion

NTM are atypical, ubiquitous environmental mycobacteria that have environmental reservoirs, including soil and both natural and municipal water supplies [1]. Diagnosis can be challenging, as NTM infection can have various clinical presentations [2]. While respiratory transmission is uncommon, particularly in immunocompetent hosts, NTM can be aerosolized and inhaled as part of steam from hot tubs or similar, causing an HP, known as “hot tub lung” [3]. Diagnosis requires clinical suspicion in conjunction with radiological and microbiological evidence, and treatment can be conservative or with corticosteroids and extended oral antibiotics, guided by a person's immune state, the organism identified, radiological findings, and burden of disease [3]. While permanent changes may occur in the lungs, in otherwise healthy individuals, long‐term outcomes are favourable.

This case highlights the importance of considering environmental sources of pulmonary injury when disease clusters are encountered.

### Disclosure Statement

Appropriate written informed consent was obtained for publication of this case report and accompanying images.
